# On Jones et al.’s method for extending Bland-Altman plots to limits of agreement with the mean for multiple observers

**DOI:** 10.1186/s12874-020-01182-w

**Published:** 2020-12-11

**Authors:** Heidi S. Christensen, Jens Borgbjerg, Lars Børty, Martin Bøgsted

**Affiliations:** 1 Department of Clinical Medicine, Aalborg University, Aalborg, Denmark; 2 Department of Haematology, Aalborg University Hospital, Aalborg, Denmark; 3 Clinical Cancer Research Center, Aalborg University Hospital, Aalborg, Denmark; 4 Department of Radiology, Aarhus University Hospital, Aarhus, Denmark

**Keywords:** Accuracy, Limits of agreement with the mean, Continuous measurements, Confidence intervals

## Abstract

**Background:**

To assess the agreement of continuous measurements between a number of observers, Jones et al. introduced limits of agreement with the mean (LOAM) for multiple observers, representing how much an individual observer can deviate from the mean measurement of all observers. Besides the graphical visualisation of LOAM, suggested by Jones et al., it is desirable to supply LOAM with confidence intervals and to extend the method to the case of multiple measurements per observer.

**Methods:**

We reformulate LOAM under the assumption the measurements follow an additive two-way random effects model. Assuming this model, we provide estimates and confidence intervals for the proposed LOAM. Further, this approach is easily extended to the case of multiple measurements per observer.

**Results:**

The proposed method is applied on two data sets to illustrate its use. Specifically, we consider agreement between measurements regarding tumour size and aortic diameter. For the latter study, three measurement methods are considered.

**Conclusions:**

The proposed LOAM and the associated confidence intervals are useful for assessing agreement between continuous measurements.

**Supplementary Information:**

The online version contains supplementary material available at 10.1186/s12874-020-01182-w.

## Background

Clinical decisions regarding diagnosis or treatment are often based on one or more measured quantities such as blood pressure, tumour size, or the diameter of an aorta. To understand the limitations of using such measurements in clinical practice, it is important to quantify how much the measurements may vary.

For almost three decades, Bland-Altman plots have been the standard method for graphical assessment of agreement between continuous measurements made by two observers or methods on a number of subjects [1]. In particular, Bland-Altman plots are often used to assess how well a new measurement method compares to a current standard method. However, if the goal is to assess the variability of measurements made by different observers it is preferable to consider more than two observers.

This prompted Jones et al. to suggest an extension of Bland-Altman’s graphical method for assessing *limits of agreement between two observers* to the *limits of agreement with the mean (LOAM) for multiple observers* [2]. Jones et al.’s LOAM have the advantage that they quantify agreement between measurements on the same scale as the measurements themselves, in contrast to the intra-class correlation (ICC) that has no unit of measure and always takes value between 0 and 1.

**Fig. 1:**
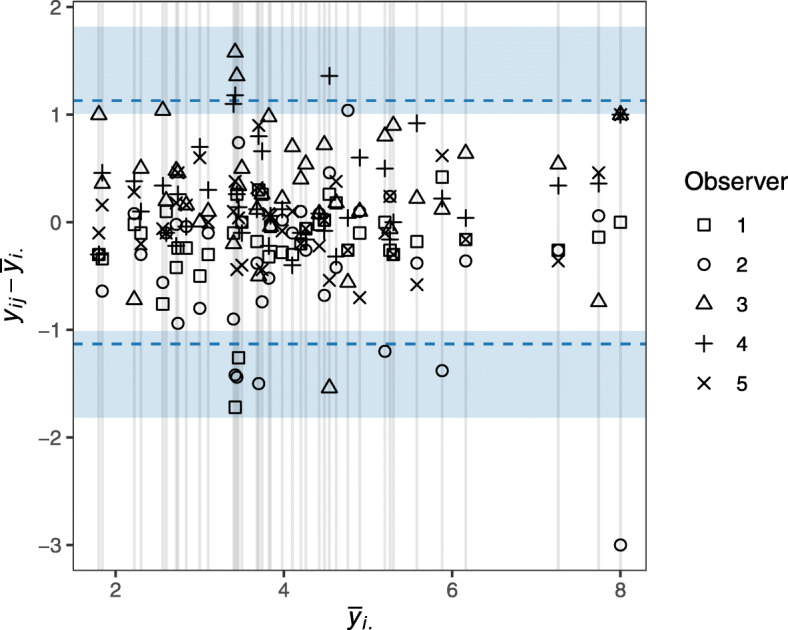
Agreement plot for tumour size measurements in centimetres with the proposed 95% LOAM (dashed line) and associated 95% CI (shading)

In more detail, consider a study where a continuous quantity is observed on *a* subjects by *b* observers (or methods). We let *y*_*ij*_ denote an observation from a random variable *Y*_*ij*_, which models the measurement performed on the *i*^th^ subject by the *j*^th^ observer for *i* = 1, …, *a* and *j* = 1, …, *b.* Assuming no preferred observer, Jones et al. suggested to assess the agreement between measurements made by different observers by investigating how much the measurements vary around the subject-specific average [2]. More formally, they were interested in how much the differences ${D}_{ij}={Y}_{ij}-{\overline{Y}}_{i\cdotp }$ are likely to vary, where ${\overline{Y}}_{i\cdotp }$ denotes the average measurement for subject *i* across the *b* observers. For visualising the data, Jones et al. propose to consider a plot of the observed differences ${d}_{ij}={y}_{ij}-{\overline{y}}_{i\cdotp }$ against the observed subject-specific average ${\overline{y}}_{i\cdotp }$. We will refer to this as an *agreement plot*. For an example of an agreement plot see Fig. 1 below. An agreement plot can, for example, help to detect whether the spread of the differences is associated to the size of the measurements, or, at least when *a* and *b* are not too large, whether some observers tend to always make large, small, or more varying measurements.

Further, Jones et al. equipped the agreement plot with horizontal lines representing the estimated 95% LOAM, which are given by ±1.96*s*, where *s* is the estimate of the residual standard deviation in a two-way analysis of variance (ANOVA) including subject and observer as fixed effects. Thus, *s* is only a measure of the residue variation left after accounting for possible subject and observer effects. On one hand, if there is a non-negligible observer effect, this should be included in the variability of the differences *d*_*ij*_ when constructing the LOAM. On the other hand, in the (unrealistic) case of no variation due to observer the 95% LOAM lines suggested by Jones et al. are biased and inefficiently estimated, as it would be custom to refit the ANOVA model without the adjustment for observer effect and adjust the degrees of freedom for *s* accordingly.

In conclusion, although the method has gained an increasing interest over the years, Jones et al. did not provide a way to: 1) assess the variation of the LOAM estimate, 2) integrate variation due to different observers, and 3) extend the method to multiple observations per observer.

In this paper, we suggest formalising Jones et al.’s approach under a simple two-way random effects model which allows us to formulate a coherent statistical inference procedure for the LOAM. In addition, we provide not only an implementation in the statistical programming software R, but also simple formulae which can be implemented in, e.g., statistical programming languages, Excel, or automatic web-modules for data collection.

## Methods

### A revised version of the limits of agreement with the mean

We propose to derive LOAM assuming a random effects model for the measurements. Assuming a statistical model provides a theoretical framework in which the LOAM can be constructed in a transparent way and furthermore enables us to supply estimates and confidence intervals (CIs) for the LOAM.

#### Statistical model

In the following we assume the measurements follow a two-way random effects model given by

1 $$ {Y}_{ij}=\mu +{A}_i+{B}_j+{E}_{ij}, $$

where *μ* describes the overall mean, and *A*_*i*_, *B*_*j*_, and *E*_*ij*_ are independent random variables following zero-mean normal distributions with variances ${\sigma}_A^2$, ${\sigma}_B^2$, and ${\sigma}_E^2$, respectively.

Under this model, measurements made by different observers are uncorrelated if they are on different subjects, while they are positively correlated with covariance ${\sigma}_A^2$ for the same subjects. Further, the covariance between measurements made by the same observer for different subjects is ${\sigma}_B^2$. Note that the measurements are assumed to be homoscedastic, i.e. has common variance, where the common variance is given by ${\sigma}_A^2+{\sigma}_B^2+{\sigma}_E^2.$ That is, the variance is split into three components: the inter-subject, inter-observer, and residual variance. Here we follow the convention of referring to the residual variance ${\sigma}_E^2$ as the intra-observer variance. Further, note that we assume a balanced data setup, where each observer has evaluated all the subjects.

#### Proposed limits of agreement with the mean

Under the two-way random effects model stated in Eq. (1), the difference between an individual measurement and the subject-specific mean, *D*_*ij*_, is normally distributed with mean zero and variance $\left({\sigma}_B^2+{\sigma}_E^2\right)\left(b-1\right)/b$. Thus, under this model we expect 95% of these differences to be within the limits

2 $$ \pm 1.96\sqrt{\frac{b-1}{b}\left({\sigma}_B^2+{\sigma}_E^2\right)}. $$

We propose the above as the 95% LOAM. To estimate ${\sigma}_B^2$ and ${\sigma}_E^2$ under the suggested two-way random effects model, we use the unbiased and consistent ANOVA estimates (see, e.g., Chapter 4 of Searle et al. [3]), given by

3 $$ {\hat{\sigma}}_B^2=\frac{MSB- MSE}{a},\kern0.5em {\hat{\sigma}}_E^2= MSE, $$

where *MSB* = *SSB*/*ν*_*B*_ and *MSE* = *SSE*/*ν*_*E*_, with $SSB=a\times {\sum}_{j=1}^b{\left({\overline{y}}_{\cdot j}-{\overline{y}}_{\cdot \cdot}\right)}^2\ \mathrm{and}\  SSE={\sum}_{i=1}^a\ {\sum}_{j=1}^b{\left({y}_{ij}-{\overline{y}}_{i\cdotp }-{\overline{y}}_{\cdotp j}+{\overline{y}}_{\cdotp \cdotp}\right)}^2$ denoting the sums of squares for the observer and residual term, and *ν*_*B*_ = *b* − 1 and *ν*_*E*_ = (*a* − 1)(*b* − 1). Further, ${\overline{y}}_{i\cdotp }$, ${\overline{y}}_{\bullet j}$, and ${\overline{y}}_{\bullet \bullet }$ denote the subject-specific, observer-specific, and overall average, respectively. Using the estimates of ${\sigma}_B^2$ and ${\sigma}_E^2$ from Eq. (3), we obtain the following estimate of the 95% LOAM:

4 $$ \pm 1.96\sqrt{\frac{SSB+ SSE}{N}}=\pm 1.96\sqrt{\frac{\sum_{i=1}^a{\sum}_{j=1}^b{\left({y}_{ij}-{\overline{y}}_{i.}\right)}^2}{N}}, $$

where *N* = *ab* is the total number of measurements. For comparison, Jones et al.’s estimate of the LOAM is given by

$$ \pm 1.96\sqrt{\frac{\sum_{i=1}^a{\sum}_{j=1}^b{\left({y}_{ij}-{\overline{y}}_{i.}-{\overline{y}}_{.j}-{\overline{y}}_{..}\right)}^2}{\nu_E}} = \pm 1.96\ {\hat{\sigma}}_E, $$

which does not include variation due to observers.

#### Confidence intervals

Instead of simply reporting the estimated LOAM given by Eq. (4), it is more informative to report CIs. However, as the distribution of the LOAM is quite complicated, we only supply approximate CIs.

Graybill and Wang propose a method for constructing (approximate) efficient CIs for linear combinations of variances [4]. To construct CIs for the LOAM in Eq. (2), we first use the method by Graybill and Wang to construct a CI for the term inside the square root of the LOAM. Next, that CI is transformed into a CI for the upper LOAM by taking the square root and then multiplying by 1.96 (see Additional file 1 for details). The resulting approximate (and asymmetric) 95% CI for the upper 95% LOAM is given by

5 $$ \left(1.96\sqrt{\left( SSB+ SSE-L\right)/N},1.96\sqrt{\left( SSB+ SSE+H\right)/N}\ \right), $$

where

$$ L=\sqrt{l_B^2{SSB}^2+{l}_E^2{SSE}^2},\kern1.75em H=\sqrt{h_B^2 SS{B}^2+{h}_E^2 SS{E}^2} $$

with ${l}_x=1-1/{F}_{0.975;{\nu}_x,\infty }$ and ${h}_x=1/{F}_{0.025;{\nu}_x,\infty }-1$ for *x* = *B* and *x* = *E* (see Graybill and Wang for other choices of *l*_*x*_ and *h*_*x*_ [4]). Here *F*_*α*; *m*, *n*_ is the *α*-quantile for the *F-*distribution with *m* numerator and *n* denominator degrees of freedom. A 95% CI for the lower 95% LOAM is simply obtained by negation of the end points of the CI for the upper LOAM, that is,

$$ \left(-1.96\sqrt{\left( SSB+ SSE+H\right)/N},-1.96\sqrt{\left( SSB+ SSE-L\right)/N}\right). $$

Simulations under the two-way random effects model in Eq. (1) indicate that the coverage probability for the approximate CI is in reality quite close to the wanted 95% even with a low number of observers (see Figure 1 in Additional file 2).

#### Sample size calculations

When planning an agreement study, it is often desirable to investigate how many measurements are necessary to obtain a certain level of precision in terms of a specified width of the CI for the LOAM. From Eq. (5) it is clear that the value of *L* and *H* determine the width of the CI for the LOAM; specifically, the CI gets narrower as *L* and *H* approaches zero. In turn, this happens when *b* is increased, since *l*_*x*_ and *h*_*x*_ approaches zero, when *ν*_*x*_ increases for both *x* = *B* and *x* = *E*. Thus, to obtain a higher precision we have to increase the number of observers, *b*, while it is not enough to increase the number of subjects.

Therefore, assume we have a fixed number of subjects *a* we want to include in a future study to assess agreement between measurements. To determine the number of observers necessary to obtain a desired width *W* of the 95% CI, we require initial estimates of ${\sigma}_B^2$ and ${\sigma}_E^2$, say ${\hat{\sigma}}_{B,0}^2$ and ${\hat{\sigma}}_{E,0}^2$, which can be obtained from, e.g., a pilot study. Exploiting the relations $SSE={\nu}_E{\hat{\sigma}}_E^2$ and $SSB={\nu}_B\times \left(a{\hat{\sigma}}_B^2+{\hat{\sigma}}_E^2\right)$, we can express the width of the CI in Eq. (5) in terms of the variance estimates rather than the sum of squares. Further, we let the estimates be given by the initial estimates ${\hat{\sigma}}_{B,0}^2$ and ${\hat{\sigma}}_{E,0}^2$, and set the width equal to *W*. That is, we want to solve the following equation with respect to *b*:

6 $$ W=\frac{1.96}{\sqrt{N}}\left(\sqrt{\ {\nu}_B\left(a{\hat{\sigma}}_{B,0}^2+{\hat{\sigma}}_{E,0}^2\right)+{\nu}_E{\hat{\sigma}}_{E,0}^2+{H}_0}-\sqrt{\ {\nu}_B\left(a{\hat{\sigma}}_{B,0}^2+{\hat{\sigma}}_{E,0}^2\right)+{\nu}_E{\hat{\sigma}}_{E,0}^2-{L}_0}\right), $$

where

7 $$ {L}_0=\sqrt{l_B^2{\nu}_B^2{\left(a{\hat{\sigma}}_{B,0}^2+{\hat{\sigma}}_{E,0}^2\right)}^2+{l}_E^2{\nu}_E^2{\left({\hat{\sigma}}_{E,0}^2\right)}^2},{H}_0=\sqrt{h_B^2{\nu}_B^2{\left(a{\hat{\sigma}}_{B,0}^2+{\hat{\sigma}}_{E,0}^2\right)}^2+{h}_E^2{\nu}_E^2{\left({\hat{\sigma}}_{E,0}^2\right)}^2}. $$

Note that *ν*_*B*_, *ν*_*E*_, *l*_*B*_, *l*_*E*_, *h*_*B*_, and *h*_*E*_ all depend on *b*. The equation can then be solved numerically with respect to *b* to find the number of observers needed to obtain an expected width *W* of the 95% CI for the 95% LOAM.

#### Inference on the variance components

In order to assess the extent of the inter-subject, inter-observer, and intra-observer variations, we suggest to consider a 95% CI for *σ*_*A*_, *σ*_*B*_, and *σ*_*E*_, respectively.

If the ANOVA estimate ${\hat{\sigma}}_B^2>0$, we simply estimate *σ*_*B*_ by ${\hat{\sigma}}_B=\sqrt{{\hat{\sigma}}_B^2}$ . Using the statistical delta method (see Additional file 3), we obtain the following approximate 95% CI for *σ*_*B*_:

8 $$ {\hat{\sigma}}_B\pm \frac{1.96}{a{\hat{\sigma}}_B}\sqrt{\frac{{\left(a{\hat{\sigma}}_B^2+{\hat{\sigma}}_E^2\right)}^2}{2{\nu}_B}+\frac{{\left({\hat{\sigma}}_E^2\right)}^2\ }{2{\nu}_E}\kern0.5em }. $$

**Fig. 2:**
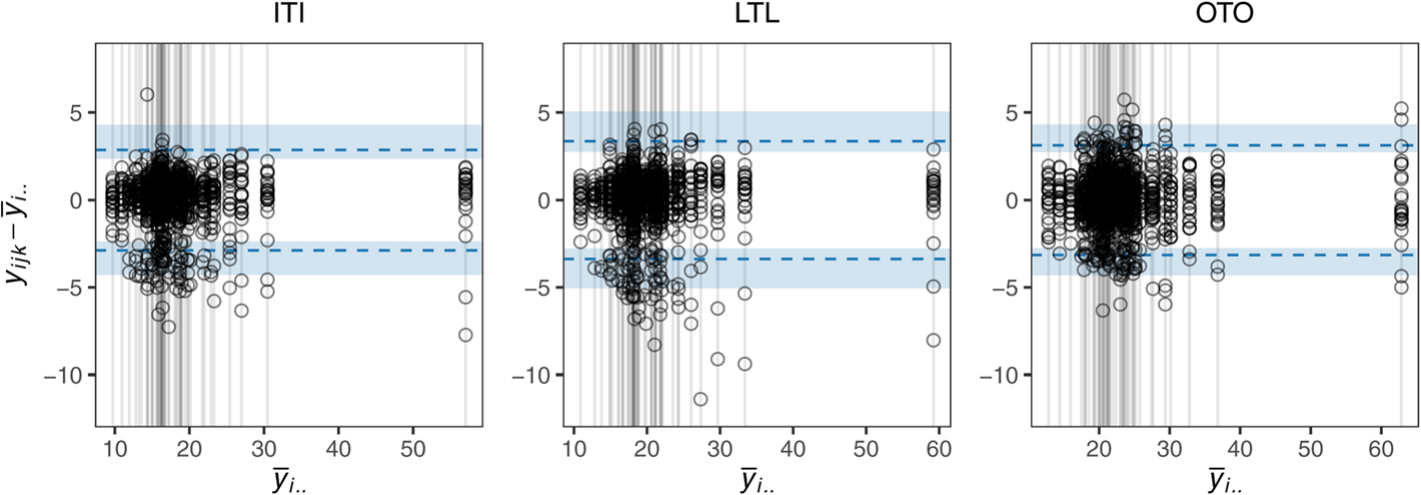
Agreement plots for each of the three methods (OTO, LTL, and ITI) used to measure the aortic diameter along with the estimate (dashed line) and the 95% CI for the 95% LOAM (shading)

Results from a small simulation study investigating how well the actual coverage of the approximate confidence interval matches the desired coverage probability and how this depends on *b* and the true values of *σ*_*B*_ and *σ*_*E*_ can be found in the additional files (see Figure 2 in Additional file 2). In general, the approximation improves as *b* increases.

It might happen the estimate ${\hat{\sigma}}_B^2$ is negative due to negative correlation between observations made by the same observer on different subjects which will indicate a misspecification of the two-way random effects model formulated in Eq. (1). Negativity can also arise by sampling variation of the unbiased ANOVA estimates, we have used in this paper. Although it is tempting to suggest setting ${\hat{\sigma}}_B^2$ to zero in such a case, this would introduce bias in the estimation. We therefore suggest to report the negative estimates, and recommend the researcher to comment on the possibility of negatively correlated measurements, and if that does not seem realistic, to assess whether the CIs are too wide to provide any clinically meaningful conclusion. It should be assessed whether more observers should be included to improve the precision of the estimate or whether the model is wrongly specified.

As the distribution of ${\hat{\sigma}}_E^2$ is known in closed form, an exact asymmetric 95% CI can easily be constructed for *σ*_*E*_ (see Additional file 3) and is given by

9 $$ \left({\hat{\sigma}}_E\sqrt{\frac{\nu_E}{\chi_{0.975;{\nu}_E}^2}},{\hat{\sigma}}_E\sqrt{\frac{\nu_E}{\chi_{0.025;{\nu}_E}^2}}\right), $$

where ${\hat{\sigma}}_E=\sqrt{{\hat{\sigma}}_E^2}$ and ${\chi}_{\alpha; {\nu}_E}^2$ is the *α*-quantile of a *χ*^2^-distribution with *ν*_*E*_ degrees of freedom.

To provide some context for the scale of ${\hat{\sigma}}_B$ and ${\hat{\sigma}}_E,$ it may also be constructive to consider ${\hat{\sigma}}_A=\sqrt{{\hat{\sigma}}_A^2}$, where ${\hat{\sigma}}_A^2=\left( MSA- MSE\right)/b$ is the ANOVA estimate of ${\sigma}_A^2$ where *MSA* = *SSA*/*ν*_*A*_ with *ν*_*A*_ = *a* − 1 and $SSA=b\ {\sum}_{i=1}^a{\left({\overline{y}}_{i\cdotp }-{\overline{y}}_{\cdotp \cdotp}\right)}^2$. The estimate of *σ*_*A*_ may be accompanied by an (approximate) 95% CI, which can be constructed using the statistical delta method (see Additional file 3):

10 $$ {\hat{\sigma}}_A\pm \frac{1.96}{b{\hat{\sigma}}_A}\sqrt{\frac{{\left(b{\hat{\sigma}}_A^2+{\hat{\sigma}}_E^2\right)}^2}{2{\nu}_A}+\frac{{\left({\hat{\sigma}}_E^2\right)}^2\ }{2{\nu}_E}\ }. $$

#### Performing an agreement analysis

To investigate agreement between observers, we propose first to make the agreement plot with the estimate and CI for the 95% LOAM from Sections 2.1.2–2.1.3, and to calculate the empirical means and standard deviations for the measurements conditional on observer or subject. Inspection of the agreement plot and the empirical means across subject, conditional on observer can be used to reveal whether any observers tend to make unusually large or small measurements. Further, the agreement plot and the conditional empirical standard deviations can be used to check whether the assumption of homoscedasticity of the random model is fulfilled. If the model in Eq. (1) is fitted using statistical software it is often possible to extract residuals and predictions of the observer and subject effects which can be used to check the model assumptions further. Specifically, one may, e.g., consider plots of the residuals against the fitted values, observer number, and subject number, respectively, to further investigate the homoscedasticity assumption. Further, a normal quantile-quantile plot of the residuals as well as of the predictions of the observer and subject effects, respectively, can be used to investigate the normality assumptions. However, if the number of observers or subjects is low, an inspection of how the predictions are distributed may be pointless. See, for example, Section 4.3 in Pinheiro and Bates for a more detailed explanation and illustration of model diagnostics [5]. If it is concluded that the model assumptions are unreasonable, one could consider an appropriate transformation of the data or formulate a variance model to handle heteroscedasticity of the outcome [5] or one could consider using a generalised, linear, and mixed model to handle non-normal distribution of outcomes [6].

If the model seems reasonable, we report the estimate and CI for the LOAM. The clinician can then compare the estimated LOAM and associated CI to a clinically acceptable difference between measurements evaluated on the same subject. Whether or not the agreement between measurements is satisfactory depends both on the scale and clinical purpose of the measurements.

Next, we may calculate CIs for *σ*_*B*_ and *σ*_*E*_, and use these along with the point estimates (${\hat{\sigma}}_B^2$ and ${\hat{\sigma}}_E^2$) to compare the order of magnitude of the inter-observer variation with the intra-observer variation. In the rare case where the observer variation is negligible, the observer effect could in principle be removed from the random model, requiring that the CIs for the LOAM are adjusted accordingly (see Additional file 4).

The agreement analysis may be supplemented with an estimate and CI for the ICC, which is another measure for agreement based on the variance components. Various forms of ICCs are listed in McGraw and Wong for a range of models [7]. The two-way random effects model proposed in this paper corresponds to Case 2A in McGraw and Wong, with subject as row effect and observer as column effect, and ICC(A, 1) can then be used to assess absolute agreement of the measurements [7]. The plug-in estimate of ICC(A, 1) is easily calculated using the estimated variance components:

$$ \hat{ICC\left(A,1\right)}=\frac{{\hat{\sigma}}_A^2}{{\hat{\sigma}}_A^2+{\hat{\sigma}}_B^2+{\hat{\sigma}}_E^2}. $$

We refer to Table 7 in McGraw and Wong for an approximate CI for ICC(A,1) [7].

### Multiple measurements on each subject per observer

The proposed LOAM and their estimates and CIs can easily be extended to the case where each observer performs multiple measurements on every subject. If each observer performs *c* measurements on each subject, we extend the two-way random effects model to:

$$ {Y}_{ijk}=\mu +{A}_i+{B}_j+{E}_{ijk}, $$

where *Y*_*ijk*_ is the *k*^th^ measurement performed by the *j*^th^ observer on the *i*^th^ subject for *i* = 1, …, *a*, *j* = 1, …, *b*, and *k* = 1, …, *c*. Note that, conditional on observer and subject, the *c* repeated measurements are assumed to be independent and identically distributed.

Mimicking the arguments for the single measurement case, but now considering the differences ${D}_{ijk}={Y}_{ijk}-{\overline{Y}}_{i\cdotp \cdotp },$ we propose the following 95% LOAM:

$$ \pm 1.96\sqrt{\frac{b-1}{b}{\sigma}_B^2+\frac{bc-1}{bc}{\sigma}_E^2}. $$

Again ${\sigma}_A^2,{\sigma}_B^2,$ and ${\sigma}_E^2$ are estimated by the ANOVA estimates (see, e.g., Chapter 4 of Searle et al. [3]), which are given by

$$ {\hat{\sigma}}_A^2=\frac{MSA- MSE}{bc},\kern0.5em {\hat{\sigma}}_B^2=\frac{MSB- MSE}{ac},\kern0.5em {\hat{\sigma}}_E^2= MSE, $$

where now *MSA* = *SSA*/*ν*_*A*_, *MSB* = *SSB*/*ν*_*B*_, and *MSE* = *SSE*/*ν*_*E*_ with $SSA= bc{\sum}_{i=1}^a{\left({\overline{y}}_{i\cdot \cdot }-{\overline{y}}_{\cdots}\right)}^2,\kern0.5em SSB= ac{\sum}_{j=1}^b{\left({\overline{y}}_{\cdot j\cdot }-{\overline{y}}_{\cdots}\right)}^2,$ $SSE={\sum}_{i=1}^a{\sum}_{j=1}^b{\sum}_{k=1}^c{\left({y}_{ijk}-{\overline{y}}_{i\cdot \cdot }-{\overline{y}}_{\cdot j\cdot }-{\overline{y}}_{\cdots}\right)}^2,$ and *ν*_*E*_ = *abc* − *a* − *b* + 1, while *ν*_*A*_ = *a* − 1 and *ν*_*B*_ = *b* − 1 is unchanged.

Note that the overall, subject-specific, and observer-specific averages (${\overline{y}}_{\cdots },{\overline{y}}_{i\cdotp \cdotp }$, and ${\overline{y}}_{\cdotp j\cdotp }$) are now also averaging across the multiple measurement index. With these definitions of *SSB*, *SSE*, *ν*_*B*_, and *ν*_*E*_ and with *N* = *abc*, the LOAM estimate and CIs still have the form given by Eq. (4)–(5). For the sample size calculation summarised in Eq. (6)–(7), we furthermore replace *a* with *ac*.

Further, CIs for *σ*_*A*_, *σ*_*B*_, and *σ*_*E*_ are obtained by Eq. (8)–(10), except that *a* is replaced with *ac*, *b* is replaced by *bc*, and the definition of ${\hat{\sigma}}_A^2,{\hat{\sigma}}_B^2,{\hat{\sigma}}_E^2,{\nu}_A,{\nu}_B$, and *ν*_*E*_ has changed to the above.

Note that all formulas for the multiple measurement case reduce to those for the single measurement case, when *c* = 1.

As for the single measurement setup, the observations may be visualised using an agreement plot, where the observed differences ${d}_{ijk}={y}_{ijk}-{\overline{y}}_{i\cdotp \cdotp }$ are plotted against the subject-specific averages ${\overline{y}}_{i\cdotp \cdotp }$.

### Data and software

The statistical programming language R, version 3.6.1 [8], was used to analyse the data in the paper. An R-package, R-scripts, and the aortic data for the LOAM calculations in the present paper can be obtained from the GitHub repository: https://github.com/HaemAalborg/loamr.

## Results

### Example 1

In a study *b* = 5 thoracic radiologists measured the diameter (in centimetres) of *a* = 40 lung tumours from computed tomography scans [9]. This study was also used as an example in Jones et al. [2]. Table 1 shows the empirical mean and standard deviation of the measurements across subject, conditional on radiologist, and Fig. 1 displays the agreement plot. Estimates and CIs of the 95% LOAM, ICC, *σ*_*A*_, *σ*_*B*_, and *σ*_*E*_ are listed in Table 2. Neither the agreement plot nor the conditional empirical mean indicate any observer systematically making unusually small or large measurements. Further, there is no indication of heteroscedasticity in relation to change in observer or to the size of the tumour.

**Table 1:** Empirical mean and standard deviation (SD) of the tumour measurements, calculated across subjects, conditional on radiologist

Radiologist	Mean (cm)	SD (cm)
1	3.9	1.6
2	3.7	1.5
3	4.4	1.6
4	4.4	1.6
5	4.1	1.6

**Table 2:** Estimates and 95% confidence intervals (CIs) of the upper 95% LOAM, intra-class correlation (ICC), *σ*_*A*_, *σ*_*B*_, and *σ*_*E*_ for the tumour measurements

LOAM (CI) in cm	ICC (CI)	${\hat{\boldsymbol{\sigma}}}_{\boldsymbol{A}}$ **(CI)** in cm	${\hat{\boldsymbol{\sigma}}}_{\boldsymbol{B}}$ **(CI)** in cm	${\hat{\boldsymbol{\sigma}}}_{\boldsymbol{E}}$ **(CI)** in cm
1.1 (1.0, 1.8)	0.8 (0.7, 0.90)	1.5 (1.1, 1.8)	0.3 (0.1, 0.5)	0.6 (0.5, 0.7)

The estimated 95% LOAM are ±1*.*1 cm (95% CI: 1.0 cm to 1.8 cm); the estimate is identical with the 95% LOAM calculated by Jones et al.’s method when rounding to one decimal place. The inter-observer standard deviation estimate is 0.3 cm (95% CI: 0.1 cm to 0.5 cm), while the intra-observer standard deviation estimate is 0.6 cm (95% CI: 0.5 cm to 0.6 cm). Although on a scale comparable to the intra-observer variation, the inter-observer variation is smaller, supporting the practice where lung nodule measurements are performed by different radiologists. We may also note that the inter-subject variation (unsurprisingly) is larger than both the inter- and intra-observer variation.

### Example 2

Borgbjerg et al. consider three methods (OTO, LTL, and ITI) for assessing the maximum antero-posterior abdominal aortic diameter [10]. A total of *b* = 12 radiologists measured the aortic diameter *c* = 2 times on *a* = 50 still abdominal aortic images to assess which of the three methods were most reliable.

Using the methods described in Section 2.2 for multiple measurements, we calculate estimates and CIs for the 95% LOAM, *σ*_*A*_, *σ*_*B*_, and *σ*_*E*_ (see Table 3) and make an agreement plot (see Fig. 2). The inter-subject variation is large compared to both the inter- and intra-observer variation. The inter-observer variation is of the same order of magnitude as the intra-observer variation and should not be excluded. The LTL method has the largest estimated LOAM, meaning that measurements made by this method tend to vary more. Conversely, the ITI method has the smallest LOAM suggesting that this method has the highest reproducibility when taking into account both the inter-observer and intra-observer variation However, the wide CIs for the LOAM indicate that more observers may be needed to assess this properly. We found significantly less intra-observer variation for the LTL and ITI compared to the OTO method. This finding is in line with the conclusion by Borgbjerg et al. which suggests that it is advantageous to employ either the ITI or LTL method when repeated measurements are performed by the same observer [10].

**Table 3:** Estimates and 95% confidence intervals (CIs) for the upper 95% LOAM, *σ*_*A*_, *σ*_*B*_, and *σ*_*E*_ for the aortic diameter measurements

Method	LOAM (CI) in mm	${\hat{\boldsymbol{\sigma}}}_{\boldsymbol{A}}$ **(CI)** in mm	${\hat{\boldsymbol{\sigma}}}_{\boldsymbol{B}}$ **(CI)** in mm	${\hat{\boldsymbol{\sigma}}}_{\boldsymbol{E}}$ **(CI)** in mm
OTO	3.2 (2.8, 4.3)	7.2 (5.7, 8.6)	1.1 (0.7, 1.6)	1.2 (1.2, 1.3)
LTL	3.4 (2.8, 5.1)	6.9 (5.5, 8.3)	1.5 (0.8, 2.1)	1.0 (1.0, 1.1)
ITI	2.9 (2.4, 4.3)	6.8 (5.4, 8.1)	1.2 (0.7, 1.8)	0.9 (0.9, 0.9)

## Discussion

In this study, we have defined the LOAM under the assumption of a two-way random effects model, with additive observer and subject effects. This allowed us to formulate a simple statistical inference procedure which can be easily implemented. The theory could be altered to cover various situations where the assumptions of the paper are not fulfilled.

First, we include observers as a random effect, meaning that we consider the observers in a study to be a random sample from a larger population of observers that we want to make inference about. It is, however, not unlikely to have a study where the considered observers constitute the whole population of interest, in which case it may be more appropriate to include observers as a fixed effect. The LOAM presented in this paper is based on the variance of the difference between an individual measurement and the subject-specific mean. Under a model with observers as fixed effect, such a LOAM will no longer measure variation due to change of observer. Depending on the purpose of the agreement study, the estimated observer effects could then be included in a reformulation of the LOAM or considered separately. However, we believe that many studies are performed to investigate agreement not only between the specific observers but rather within a larger population of observers, encouraging the choice of model in this paper.

Second, one could imagine a situation where it is relevant to include an interaction term between subjects and observers, that is, modelling that observers may react differently upon the subjects. For single measurements this interaction effect is confounded with the residual error, but for multiple measurements this effect could in principle be modelled and the LOAM adjusted accordingly.

Third, the methods and formulae of this paper rely on the assumption of a balanced data setup, where all observers have evaluated all the subjects the same number of times. However, in practice it is not unlikely to encounter an unbalanced data set as measurements may get lost or not all observers were able to perform all measurements. An unbalanced setup is definitely more complicated to handle but some advances can be made. A new expression for the LOAM may be found under a two-way random model allowing unbalanced data, while existing methods for finding estimates of the variance components can be used to estimate the adjusted LOAM (see, e.g., [3, 11]). However, it is in general not possible to obtain closed form expressions for the confidence intervals for the LOAM and variance components.

Fourth, as indicated in Section 2.1.5 it might happen that the estimate ${\hat{\sigma}}_B^2$ is negative due to negative correlation between observations made by the same observer on different subjects which will indicate a misspecification of the two-way random effects model formulated in Eq. (1). It is possible to generalise the theory by considering marginal modelling [12]. It was further indicated in Section 2.1.5 that negativity can also arise by sampling variation of the unbiased ANOVA estimates, we have used in this paper. Various approaches have been suggested to remedy this problem as well [13].

Pursuing these generalisations will, however, make modelling and implementation much more involved, and thereby violate our goal to formulate an easily implementable framework.

## Conclusions

Our results show it is possible to formulate measures for the agreement with the mean between multiple observers, equip them with confidence intervals, and extend them to multiple observations per observer, thereby providing a natural extension of Bland-Altman’s graphical method. We believe, we have provided an easily accessible and useful statistical toolbox for researchers involved in assessing agreement between methods or individuals performing clinical measurements.

## Supplementary Information

**Additional file 1.** Derivation of the confidence intervals for the LOAM.

**Additional file 2.** Coverage probabilities from a small simulation study.

**Additional file 3.** Derivation of confidence intervals for the variance parameters.

**Additional file 4.** Formulae after removing the observer effect.

## Data Availability

The dataset on abdominal aortic diameter measurements supporting the conclusions of this article is available in the *loamr* repository: https://github.com/HaemAalborg/loamr. The dataset on tumour sizes is not publicly available but is available from the corresponding author of the original paper on request [9].

## Abbreviations

LOAM: Limits of agreement with the mean
ICC: Inter-class correlation
ANOVA: Analysis of variance
CI: Confidence interval

## References

[1] Bland JM, Altman DG (1986). Statistical methods for assessing agreement between two methods of clinical measurement. Lancet.

[2] Jones M, Dobson A, O’brian S (2011). A graphical method for assessing agreement with the mean between multiple observers using continuous measures. Int J Epidemiol.

[3] Searle SR, Casella G, McCulloch CE (1992). Variance Components.

[4] Graybill FA, Wang C-M (1980). Confidence intervals on nonnegative linear combinations of variances. J Am Stat Assoc.

[5] Pinheiro JC, Bates DM (2000). Mixed-effects models in S and S-PLUS.

[6] McCulloch CE, Searle SR, Neuhaus JM (2008). Generalized, Linear, and Mixed Models.

[7] McGraw KO, Wong SP (1996). Forming inferences about some intraclass correlation coefficients. Psychol Methods.

[8] R Core Team (2019). “R: A Language and Environment for Statistical Computing.” Vienna, Austria.

[9] Erasmus JJ (2003). Interobserver and intraobserver variability in measurement of non-small-cell carcinoma lung lesions: implications for assessment of tumor response. J Clin Oncol.

[10] Borgbjerg J, Bøgsted M, Lindholt JS, Behr-Rasmussen C, Hørlyck A, Frøkjær JB (2018). Superior reproducibility of the leading to leading edge and inner to inner edge methods in the ultrasound assessment of maximum abdominal aortic diameter. Eur J Vasc Endovasc Surg.

[11] Burdick RK, Borror CM, Montgomery DC (2005). Design and Analysis of Gauge R&R Studies: Making Decisions with Confidence Intervals in Random and Mixed ANOVA Models,. SIAM, Philadelphia.

[12] G. Mohlenberghs and G. Verbeke, “A note on a hierarchical interpretation for negative variance components,” Stat. Modelling, vol. 11, no. 5, pp. 389–408, doi: 10.1177/1471082X1001100501.

[13] André I. Khuri, “Designs for Variance Components Estimation: Past and Present,” Int. Stat. Rev*.*, vol. 68, no. 3, pp. 311–322, doi: 10.1111/j.1751-5823.2000.tb00333.x.

