# Supplementary material for: On Jones et al.’s method for extending Bland-Altman plots to limits of agreement with the mean for multiple observers
Source: BMC Med Res Methodol. 2020 Dec 11;20:304. doi: 10.1186/s12874-020-01182-w (PMC7730774; doi:10.1186/s12874-020-01182-w)
Supplement: Supplementary file 1 — Additional file 1. Derivation of the confidence intervals for the LOAM. [file 12874_2020_1182_MOESM1_ESM.docx]

**Additional file 1: Derivation of the confidence intervals for the LOAM**

In the following we show how the confidence intervals for the 95% LOAM have been constructed. The calculations are performed for the model for multiple measurements per observer from Section 2.2 in the paper, but they can easily be reduced to the case of single measurements by letting $c=1.$

Recall that the upper 95% LOAM is given by

$$1.96\sqrt{\frac{b-1}{b} \sigma_{B}^{2}+\frac{bc-1}{bc}\sigma_{E}^{2}}.$$

We let $\theta_{B}$ and $\theta_{E}$ denote the expectation of the mean sum of squares for the observer and residual term, respectively, that is, $\theta_{B}=E\left[ MSB \right]=ac\sigma_{B}^{2}+\sigma_{E}^{2}$ and $\theta_{E}=E\left[ MSE \right]=\sigma_{E}^{2}$. Cf. Searle al. [1] we have that

$$\frac{\nu_{B}}{\theta_{B}}MSB\sim\chi_{\nu_{B}}^{2} is independent of \frac{\nu_{E}}{\theta_{E}}MSE\sim\chi_{\nu_{A}}^{2},$$

where $\nu_{B}=b-1, \nu_{A}=abc-a-b+1,$ and $\chi_{\nu}^{2}$ denotes the $\chi^{2}$-distribution with $\nu$ degrees of freedom.

Thus, we can use results from Graybill and Wang to find a confidence interval for any nonnegative linear combination of $\theta_{B}$ and $\theta_{E}$ [2]. Consider constants $K_{B}$ and $K_{E}$ such that

$$K_{B}\theta_{B}+K_{E}\theta_{E}= \frac{b-1}{b} \sigma_{B}^{2}+\frac{bc-1}{bc}\sigma_{E}^{2},$$

that is, $K_{B}= \frac{b-1}{abc}$ and $K_{E}= \frac{abc-a-b+1}{abc}$. Then an approximate 95% confidence interval for this linear combination is obtained by Eq. (2.2) in Graybill and Wang [2]:

$$\left( K_{B}MSB+K_{E}MSE-\sqrt{K_{B}^{2}l_{B}^{2}MSB^{2}+K_{E}^{2}l_{E}^{2}MSE^{2}} , K_{B}MSB+K_{E}MSE+\sqrt{K_{B}^{2}h_{B}^{2}MSB^{2}+K_{E}^{2}h_{E}^{2}MSE^{2}} \right),$$

where $l_{x}= 1-1/F_{0.975; \nu_{x}, \infty}$ and $h_{x}= 1/F_{0.025; \nu_{x}, \infty}-1$ for $x=B$ and $x=E$ (see Graybill and Wang for other choices of $l_{x}$ and $h_{x}$ [2]). Here $F_{\alpha;m, n}$is the *α*-quantile for the *F-*distribution with *m* numerator and *n* denominator degrees of freedom. The above confidence interval can also be expressed as

$$\left( \frac{1}{abc}\left( SSB+SSE-\sqrt{l_{B}^{2}SSB^{2}+l_{E}^{2}SSE^{2}} \right), \frac{1}{abc}\left( SSB+SSE+\sqrt{h_{B}^{2}SSB^{2}+h_{E}^{2}SSE^{2}} \right) \right).$$

An approximate 95% confidence interval for the upper 95% LOAM can now be obtained by transforming the endpoints, that is, taking the square root followed by multiplication with 1.96.

**References**

[1] S. R. Searle, G. Casella, and C. E. McCulloch, *Variance Components*. Hoboken: John Wiley & Sons, Inc., 1992.

[2] F. A. Graybill and C.-M. Wang, “Confidence intervals on nonnegative linear combinations of variances,” *J. Am. Stat. Assoc.*, vol. 75, no. 372, pp. 869–873, Dec. 1980.
