# Supplementary material for: On Jones et al.’s method for extending Bland-Altman plots to limits of agreement with the mean for multiple observers
Source: BMC Med Res Methodol. 2020 Dec 11;20:304. doi: 10.1186/s12874-020-01182-w (PMC7730774; doi:10.1186/s12874-020-01182-w)
Supplement: Supplementary file 2 — Additional file 2. Coverage probabilities from a small simulation study. [file 12874_2020_1182_MOESM2_ESM.docx]

**Additional file 2: Coverage probabilities from a small simulation study**

**Estimated coverage for the approximate confidence interval for the upper LOAM**


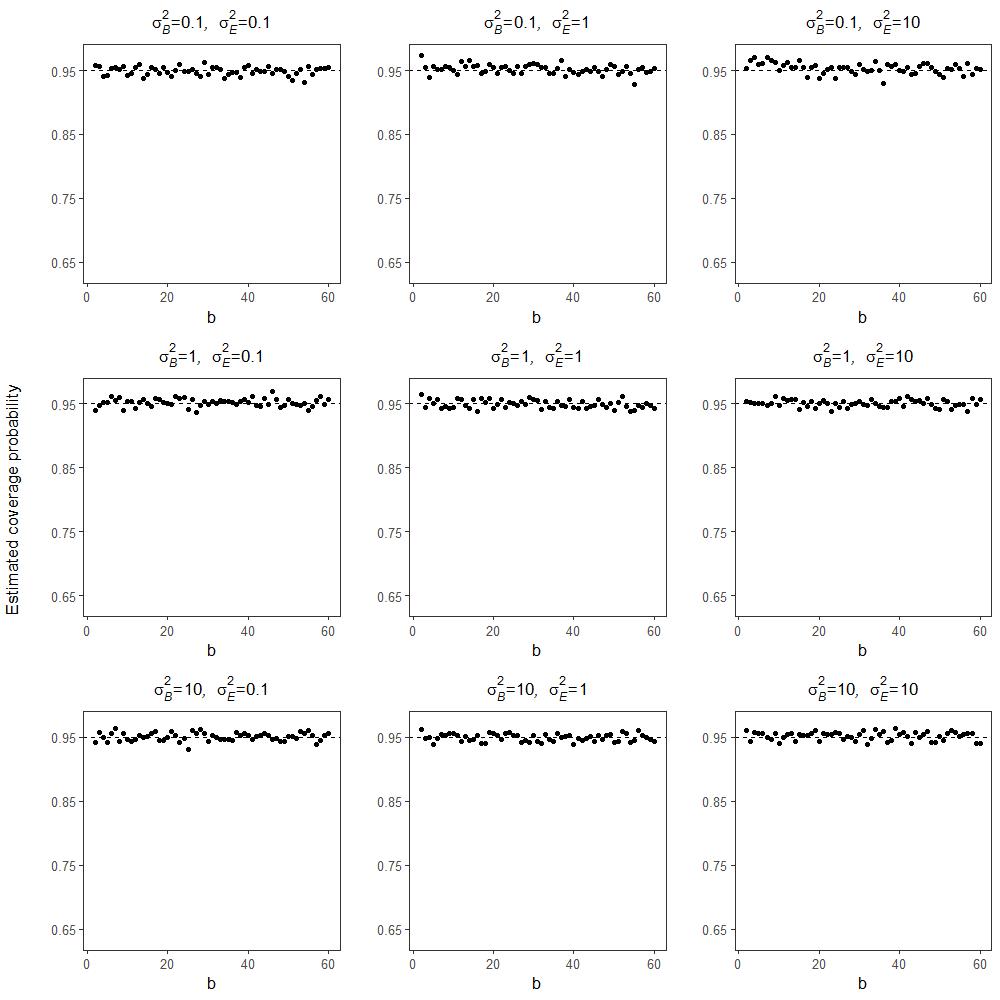


Figure 1. Estimated coverage probability for the approximate 95% confidence interval for the upper LOAM stated in Eq. (5) in the paper. The estimation is based on 1000 simulations from the two-way random effects model with $a=10, c=1, \mu=0$, $\sigma_{A}^{2}=5$, while $b=2,\ldots, 60$ and nine combinations of $\sigma_{B}^{2}$ and $\sigma_{E}^{2}$ are considered (see specific choices in the title of each plot). The estimated coverage probability is close to the wanted 95% for all the considered parameter values and all values of $b$.

**Estimated coverage for the approximate confidence interval for** $\boldsymbol{\sigma}_{\boldsymbol{B}}$


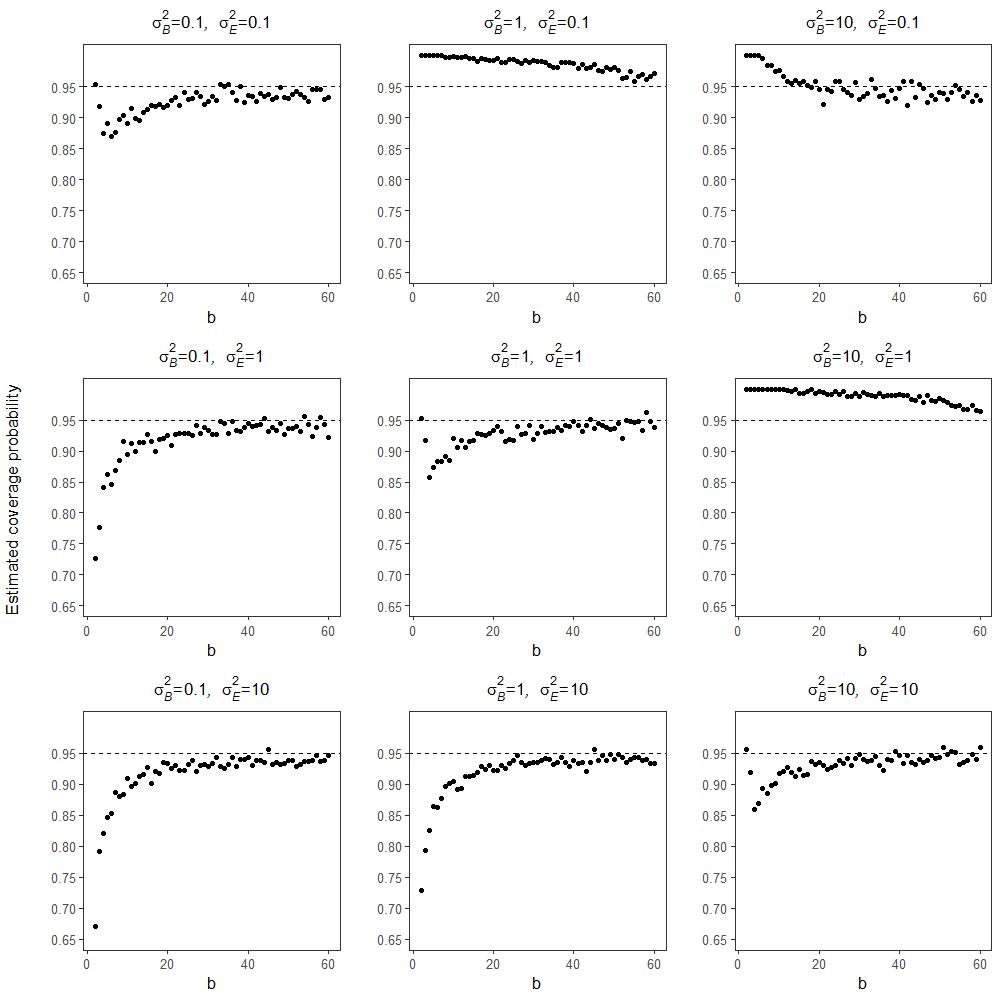


Figure 2. Estimated coverage probability for the approximate 95% confidence interval for $\sigma_{B}$ stated in Eq. (8) in the paper. The estimation is based on 1000 simulations from the two-way random effects model with $a=10, c=1, \mu=0$, $\sigma_{A}^{2}=5$, while $b=2,\ldots, 60$ and nine combinations of $\sigma_{B}^{2}$ and $\sigma_{E}^{2}$ are considered (see specific choices in the title of each plot). In general, for $b>20$ the estimated coverage probability is close to the wanted 95%, while the approximation may be poor for small values of $b$.

**Estimated coverage for the approximate confidence interval for** $\boldsymbol{\sigma}_{\boldsymbol{E}}$


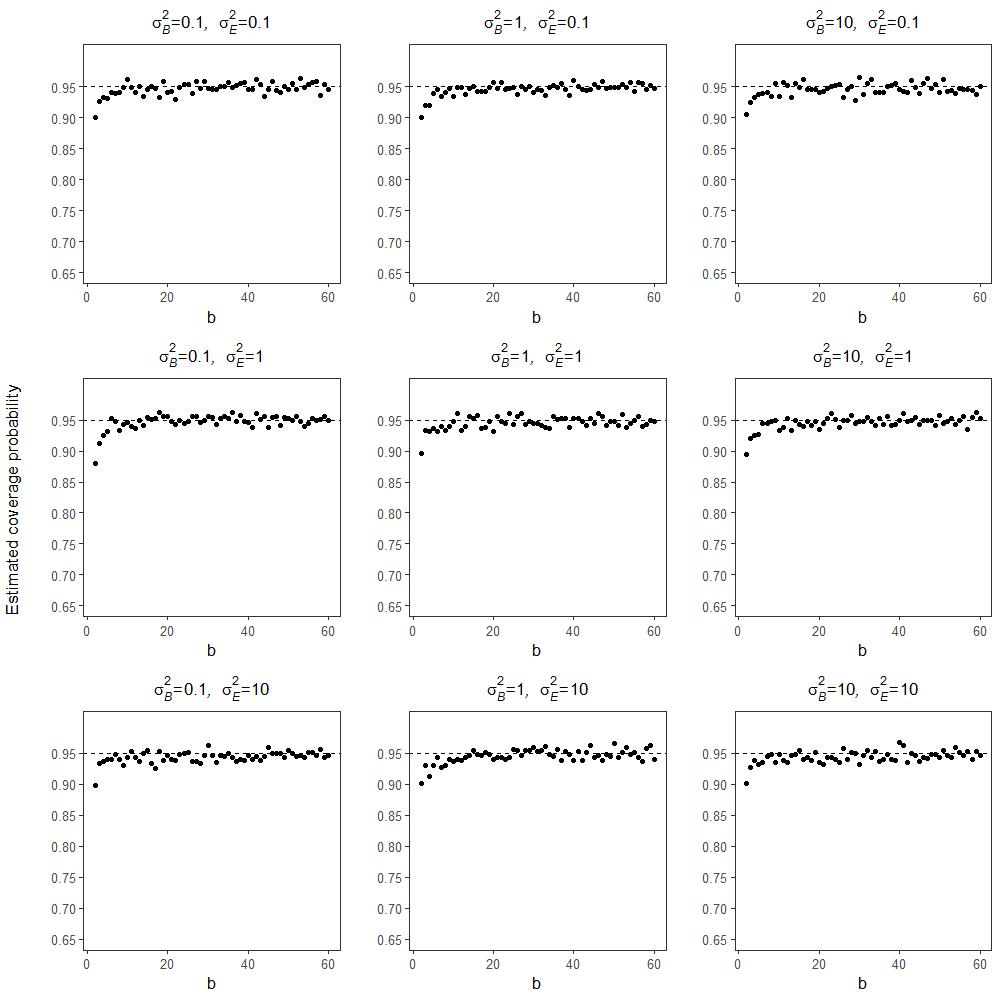


Figure 3. Estimated coverage probability for the symmetric and approximate 95% confidence interval for $\sigma_{E}$ [see Additional file 3]. The estimation is based on 1000 simulations from the two-way random effects model with $a=10, c=1, \mu=0$, $\sigma_{A}^{2}=5$, while $b=2,\ldots, 60$ and nine combinations of $\sigma_{B}^{2}$ and $\sigma_{E}^{2}$ are considered (see specific choices in the title of each plot). In general, the estimated coverage probability is close to the wanted 95%, perhaps except for small values of $b$ ($b<5)$. Note that an exact confidence interval for $\sigma_{E}$is supplied in the main paper.
