# Supplementary material for: On Jones et al.’s method for extending Bland-Altman plots to limits of agreement with the mean for multiple observers
Source: BMC Med Res Methodol. 2020 Dec 11;20:304. doi: 10.1186/s12874-020-01182-w (PMC7730774; doi:10.1186/s12874-020-01182-w)
Supplement: Supplementary file 3 — Additional file 3. Derivation of confidence intervals for the variance parameters. [file 12874_2020_1182_MOESM3_ESM.docx]

**Additional file 3: Derivation of confidence intervals for the variance parameters**

The following describes how confidence intervals can be constructed for the variance parameters $\sigma_{A}, \sigma_{B}$, and $\sigma_{E}$ in the two-way random model. Here we consider the model stated in Eq. (1) in the main paper, but the calculations are straightforwardly extended to the setup in Section 2.2 in the main paper, where each observer performs multiple measurements on the same subject.

**Confidence interval for** $\boldsymbol{\sigma}_{\boldsymbol{B}}$**:**

Let $\chi_{b-1}^{2}$ denote the $\chi^{2}$-distribution with $b-1$ degrees of freedom, $N\left( \mu, \sigma^{2} \right)$ the normal distribution with mean $\mu$ and variance $\sigma^{2}$, and $\underset{\to}{D}$ convergence in distribution.

We have cf. Searle et al [1] that

$$\frac{\left( b-1 \right)MSB}{a\sigma_{B}^{2}+\sigma_{E}^{2}}\sim\chi_{b-1}^{2},$$

which converges to a $N\left( b-1, 2\left( b-1 \right) \right)$-distribution when $b\to\infty$. This entails that

$$MSB \underset{\to}{D} N\left( a\sigma_{B}^{2}+\sigma_{E}^{2}, \frac{2\left( a\sigma_{B}^{2}+\sigma_{E}^{2} \right)^{2}}{b-1} \right) as b\to\infty.$$

Similarly, we obtain that

$$MSE \underset{\to}{D} N\left( \sigma_{E}^{2}, \frac{2\left( \sigma_{E}^{2} \right)^{2}}{(a-1)(b-1)} \right) as ab\to\infty.$$

As $MSB$ and $MSE$are independent [1], we have that

$$\hat{\sigma}_{B}^{2}= \frac{MSB-MSE}{a} \underset{\to}{D} N\left( \sigma_{B}^{2}, \frac{2}{a^{2}}\left( \frac{\left( a\sigma_{B}^{2}+\sigma_{E}^{2} \right)^{2}}{\left( a-1 \right)\left( b-1 \right)}+\frac{\left( \sigma_{E}^{2} \right)^{2}}{b-1} \right) \right) as a,b\to\infty.$$

To find the asymptotic distribution of $\hat{\sigma}_{B}=\sqrt{\hat{\sigma}_{B}^{2}}$, we use the statistical delta method for the transformation $g\left( x \right)=\sqrt{x}$ (see, e.g., [2]) . This gives us

$$\hat{\sigma}_{B} \underset{\to}{D} N\left( \sigma_{B}, \frac{1}{{2a}^{2}\sigma_{B}^{2}}\left( \frac{\left( a\sigma_{B}^{2}+\sigma_{E}^{2} \right)^{2}}{\left( a-1 \right)\left( b-1 \right)}+\frac{\left( \sigma_{E}^{2} \right)^{2}}{b-1} \right) \right) as a,b\to\infty,$$

from which an approximate confidence interval can be obtained for $\sigma_{B}$ using plug-in estimates of the variance components. Figure 2 in Additional file 2 displays results from a small simulation study on the accuracy of this approximate confidence interval.

**Confidence interval for** $\boldsymbol{\sigma}_{\boldsymbol{A}}$**:**

An approximate confidence interval for $\sigma_{A}$ is obtained by mimicking the arguments for $\sigma_{B}$, that is, one simply needs to interchange the role of $a$ and $b$ as well as substitute $\sigma_{B}$ with $\sigma_{A}$ and $MSB$ with $MSA$.

**Confidence intervals for** $\boldsymbol{\sigma}_{\boldsymbol{E}}$**:**

We have cf. Searle et al [1] that

$$\frac{\nu_{E}MSE}{\sigma_{E}^{2}}\sim\chi_{\nu_{E}}^{2},$$

where $\nu_{E}=(a-1)(b-1)$. From this we can straightforwardly calculate an exact confidence interval for $\sigma_{E}^{2}$, which in turn can be transformed into an exact confidence interval for $\sigma_{E}.$ The resulting 95% confidence interval for $\sigma_{E}$ is given by

$$\left( \hat{\sigma}_{E}\sqrt{\frac{\nu_{E}}{\chi_{0.975;\nu_{E}}^{2}}}, \hat{\sigma}_{E}\sqrt{\frac{\nu_{E}}{\chi_{0.025; \nu_{E}}^{2}}} \right),$$

where $\chi_{\alpha;\nu_{E}}^{2}$ is the $\alpha$-quantile of a $\chi^{2}$-distribution with $\nu_{E}$ degrees of freedom.

Alternatively, an approximate (symmetric) confidence interval can be constructed in a similar manner to the confidence interval for $\sigma_{B}$ using the delta method. The resulting approximate 95% confidence interval is given by

$$\hat{\sigma}_{E}\pm1.96\frac{\hat{\sigma}_{E}}{\sqrt{2\nu_{E}}} .$$

Figure 3 in Additional file 2 displays results from a small simulation study on the accuracy of this approximate confidence interval.

**References**

[1] S. R. Searle, G. Casella, and C. E. McCulloch, *Variance Components*. Hoboken: John Wiley & Sons, Inc., 1992.

[2] A. W. van der Vaart, *Asymptotic statistics*. Cambridge: Cambridge University Press, 1998.
