# Supplementary material for: On Jones et al.’s method for extending Bland-Altman plots to limits of agreement with the mean for multiple observers
Source: BMC Med Res Methodol. 2020 Dec 11;20:304. doi: 10.1186/s12874-020-01182-w (PMC7730774; doi:10.1186/s12874-020-01182-w)
Supplement: Supplementary file 4 — Additional file 4. Formulae after removing the observer effect. [file 12874_2020_1182_MOESM4_ESM.docx]

**Additional file 4: Formulae after removing the observer effect**

If an agreement analysis has been performed (following the cookbook of Section 2.1.6 in the main paper), and the observer variation has been deemed negligible, then the observer effect should be removed from the random model for the measurements, and formulae for the LOAM, confidence intervals, and estimates should be adjusted accordingly. In the following, we restate the relevant formulae under the one-way random model with subject effect only. That is, we assume the measurements now follow the model

$$Y_{ijk}=\mu+A_{i}+ W_{ijk},$$

where $\mu$ is the overall mean, and $A_{i}$ and $W_{ijk}$ are random variables following zero-mean normal distributions with variances $\sigma_{A}^{2}$ and $\sigma_{W}^{2}$, respectively, for $i=1, \ldots, a$, $j=1,\ldots, b$, and $k=1,\ldots, c$. Note that we (as opposed to the main paper) immediately consider the model for the case with multiple measurements per observer. In the case of only one observation per observer per subject, this is the special case with $c=1$.

Assuming the above model, the difference, $D_{ijk}=Y_{ijk}-\bar{Y}_{i\cdot\cdot}$, has variance $\frac{bc-1}{bc}\sigma_{W}^{2}$, and the LOAM becomes

$$\pm1.96\sqrt{\frac{bc-1}{bc}\sigma_{W}^{2}}.$$

The maximum likelihood/ANOVA estimate of $\sigma_{W}^{2}$ is given by (see e.g. Chapter 4 of Searle et al. [1])

$$\hat{\sigma}_{W}^{2}=MSW,$$

where $MSW= SSW/\nu_{W}$ for $SSW= \sum_{i=1}^{a} \sum_{j=1}^{b} \sum_{k=1}^{c} \left( y_{ijk}- \bar{y}_{i\cdot\cdot} \right)^{2}$and $\nu_{W}=a(bc-1)$. The LOAM can then be estimated by

$$\pm1.96\sqrt{\frac{SSW}{abc}.}$$

Note that this estimate is identical to the one in the main paper as $SSW=SSB+SSE$, while the following confidence intervals are not.

An exact asymmetric confidence interval is easily found for $\sigma_{W}$ by utilizing that $\hat{\sigma}_{W}^{2}$ follows a scaled $\chi^{2}$-distribution with $\nu_{W}$ degrees of freedom (see e.g. Chapter 4 of Searle et al. [1]). This can then be transformed into the following exact 95% confidence interval for the LOAM:

$$\left( 1.96\sqrt{\frac{\left( bc-1 \right)SSW}{bc \chi_{0.975;\nu_{W}}^{2}}}, 1.96\sqrt{\frac{\left( bc-1 \right)SSW}{bc \chi_{0.025;\nu_{W}}^{2}}} \right),$$

where $\chi_{\alpha;\nu_{W}}^{2}$ is the $\alpha$-quantile for the $\chi^{2}$-distribution with $\nu_{W}$ degrees of freedom.

**References**

[1] S. R. Searle, G. Casella, and C. E. McCulloch, *Variance Components*. Hoboken: John Wiley & Sons, Inc., 1992.
